# Shiga toxin-producing *Escherichia coli* (STEC) and atypical enteropathogenic *E. coli* (aEPEC) in Swedish retail wheat flour

**DOI:** 10.1099/acmi.0.000577.v3

**Published:** 2023-05-03

**Authors:** Robert Söderlund, Catarina Flink, Anna Aspán, Erik Eriksson

**Affiliations:** ^1^​ Department of Microbiology, Swedish National Veterinary Institute (SVA), Uppsala, Sweden; ^2^​ Department of Biology, Swedish Food Agency, Uppsala, Sweden

**Keywords:** food safety, food microbiology, foodborne infections, genomics, zoonosis, cereals, One Health, EHEC

## Abstract

Wheat flour has been identified as the source of multiple outbreaks of gastrointestinal disease caused by shiga toxin-producing *Escherichia coli* (STEC). We have investigated the presence and genomic characteristics of STEC and related atypical enteropathogenic *E. coli* (aEPEC) in 200 bags of Swedish-produced retail wheat flour, representing 87 products and 25 brands. Samples were enriched in modified tryptone soya broth (mTSB) and screened with real-time PCR targeting *stx_1_*, *stx_2_* and *eae*, and the serogroups O157, O121 and O26. Isolation was performed by immunomagnetic separation (IMS) for suspected STEC/aEPEC O157, O121 and O26, and by screening pools of colonies for other STEC. Real-time PCR after enrichment revealed 12 % of samples to be positive for shiga toxin genes (*stx_1_* and/or *stx_2_*) and 11 % to be positive for intimin (*eae*). Organic production, small-scale production or whole grain did not significantly influence shiga toxin gene presence or absence in a generalized linear mixed model analysis. Eight isolates of STEC were recovered, all of which were intimin-negative. Multiple serotype/sequence type/shiga toxin subtype combinations that have also been found in flour samples in other European countries were recovered. Most STEC types recovered were associated with sporadic cases of STEC among humans in Sweden, but no types known to have caused outbreaks or severe cases of disease (i.e. haemolytic uraemic syndrome) were found. The most common finding was O187:H28 ST200 with *stx_2g_*, with possible links to cervid hosts. Wildlife associated with crop damage is a plausible explanation for at least some of the surprisingly high frequency of STEC in wheat flour.

## Data Summary

Whole-genome sequence data from STEC and aEPEC isolates are available from the European Nucleotide Archive (ENA) under project number PRJEB56696. Sample metadata and analysis results are presented in File S1, available in the online version of this article.

## Introduction

Shiga toxin-producing *Escherichia coli* (STEC) are zoonotic gastrointestinal pathogens capable of causing mild to severe diarrhoea with a risk of potentially life-threatening complications, including haemolytic uraemic syndrome (HUS) [1]. Severe infections with HUS are more common among young children [2]. STEC colonize the intestines of warm-blooded animals and particularly ruminants, usually without causing clinical signs, but can also be transmitted by various other animals [3]. Transmission to humans occurs via the faecal–oral route, and contaminated beef, dairy products and vegetables such as leafy greens are frequently implicated in outbreaks [4]. The serotypes O157:H7, O26:H11 and O121:H19 are commonly associated with severe human infection in Sweden [5] and many other countries, but severe cases can be caused by a wide range of other serotypes [6]. A related pathotype in terms of virulence attributes is enteropathogenic *E. coli* (EPEC), characterized by the locus of enterocyte attachment and effacement (LEE), which is also frequently present in STEC associated with severe disease, but lacking genes encoding shiga toxins [7]. EPEC infection causes diarrhoea in humans, particularly infants [8], but is not associated with HUS. EPEC with the *E. coli* adherence factor plasmid are referred to as typical EPEC (tEPEC) and those without are considered atypical EPEC (aEPEC) [7]. Certain aEPEC are closely related to STEC and may represent strains that have never acquired *stx* genes or be the result of *stx* gene loss. In animal reservoirs the *stx*-negative forms of such combined aEPEC/STEC lineages can be more common for certain serovars [9].

In recent years multiple STEC outbreaks have been described in which undercooked flour has been identified as the source. The first of these was a multi-state outbreak of O157:H7 in the USA in 2009, epidemiologically linked to consumption of commercial pre-packaged cookie dough [10]. This was followed by outbreaks of O121 and O26 linked to flour in the USA in 2016 [11], O121:H19 in Canada 2016–2017 [12] and O26:H11 again in the USA in 2019 [13], with cases in all these outbreaks reporting eating raw dough and the outbreak strains linked to isolates from flour samples by whole-genome sequencing. An additional smaller outbreak of O157:H7 in the USA was linked to eating dessert pizza made from proprietary dough mix in 2016, but in this case no matching strain could be isolated from food samples [14]. In 2022, an outbreak of STEC O26 linked to consumption of undercooked frozen pizza dough in France caused several cases of severe disease and two deaths [15, 16]. Previous experience of flour as a source appears to have been a key factor in the successful investigation of some of these outbreaks, raising the question of whether flour has been overlooked as a potential source of outbreaks and sporadic STEC cases worldwide. A few studies have been performed analysing the presence of STEC at various stages of the production chain in different countries. Of 51 wheat and rye flour samples collected in German production facilities 2014–2017, 39 % were positive by PCR after enrichment, and STEC was successfully isolated from 19 % [17]. The isolates were not characterized. Two Swiss studies successfully isolated STEC from 11 % of 70 retail flour samples [18] and 9 % of 93 samples [19] respectively. The recovered isolates included the known human-pathogenic serotypes O26:H11 and O103:H2. A larger, more recent study performed in Canada isolated STEC from 1.7 % of 347 retail flour samples and recovered a likely pathogenic strain of O103:H25 [20].

In the present study we have analysed 200 Swedish retail wheat flour samples and attempted to identify risk factors in terms of product type and source for the presence of STEC. We have also characterized all recovered isolates of STEC and EPEC by whole-genome sequencing.

## Methods

### Samples and metadata

A total of 200 retail bags of flour made from Swedish-grown wheat were purchased 2020–2021. Products were classified as whole wheat flour or white flour, from conventional or organic production, and as from a large/medium or small brand, with the former defined as any brand with nationwide distribution, including the own-brands of supermarket chains. The samples represented 87 products from 8 large/medium and 17 small brands. Multiple brands, in particular among the medium and large ones, likely source their flour from the same mills. All replicates of the same product were purchased on separate occasions and had different batch numbers or different use-by dates if no batch number was present. Of the 87 products, 24 were analysed in triplicate and 13 common standard products in double triplicate, whereas 50 less common products were analysed without replication. Full sample data are presented in File S1 (available in the online version of this article). From each bag, approximately 100 g was carefully removed and stored in a sealed plastic sample jar in a climate-controlled room (~20 °C) until analysed. The analysed sample quantities were based on volume instead of weight to minimize handling and thereby avoid cross-contamination from dust. Three blank jars handled in parallel were included as negative controls.

### Enrichment and real-time PCR screening

Each jar of ~100 g flour was emptied into a bag with Oxoid modified soya broth (mTSB) with 4 g l^−1^ dipotassium hydrogen phosphate and 1.5 g l^−1^ bile salts at a ratio of 1 : 3, mixed and incubated 16–20 h at 37 °C. Aliquots from the mixed enriched samples were allowed to sediment in 50 ml tubes until a semi-clear supernatant formed. Approximately 1 ml of this supernatant was heated to 95 °C for 5 min, cooled, mixed and centrifuged for 2 min at 10 000 r.f.c., and 200 µl of the resulting lysate was used for DNA extraction in a Qiagen EZ1 BioRobot with the Blood and Tissue kit. Real-time PCR was performed using an ABI7500 instrument with 15 µl reactions (including 2 µl template) of Quantabio PerfeCTa ToughMix. Each sample was analysed using two triplex reactions targeting (i) O157 [21], O26 [21] and O103 [22] and (ii) *stx_1_*, *stx_2_* (shiga toxin 1/2) [21] and *eae* (intimin) [23] . Any sample with *C*_t_<36 was considered positive. DNA extractions from blank control samples were analysed and confirmed negative, and each run included positive and negative template controls.

### Isolation of STEC and EPEC

Samples that were found to be positive for O157, O26 or O121 by PCR were further investigated by immunomagnetic separation (IMS) using Thermo Fisher Dynabeads EPEC/VTEC kits to isolate strains of STEC or EPEC of these serotypes. To isolate all other STEC, samples were selected that had *C*_t_<32 for either *stx_1_* or *stx_2_*. From these enriched mTSB cultures, pools of 5×10 colonies consistent with *E. coli* when grown on Oxoid tryptone bile x-glucuronide agar (TBX) plates were prepared and analysed by real-time PCR. From *stx*-positive pools 10 individual colonies were cultured and analysed in the same way, identifying any STEC, which were then confirmed by repeating the PCR.

### Whole-genome sequencing and bioinformatics

DNA was extracted from all recovered isolates using an EZ1 robot as described above, quantified using the Thermo Fisher Qubit BR kit and submitted for sequencing as paired-end 2×150 bp with Nextera library preparation and using an Illumina NovaSeq at the SciLifeLab Clinical Genomics facility, Solna, Sweden. All isolates were sequenced to a minimum of 100× coverage. Serotyping *in silico* [24], virulence gene detection [25] and multi-locus sequence typing (MLST) [26] were performed with CGE online tools (http://www.genomicepidemiology.org/). Sequence data were uploaded to the European Nucleotide Archive (ENA) and are available under project number PRJEB56696.

### Statistical analysis

The individual product was included as a random effect to account for replication in a generalized linear mixed-effects model (GLMM) performed using the glmer function of the lme4 package in R 3.6.0, testing the above-mentioned binary variables against PCR outcomes (any *stx*, i.e. *stx_1_* or *stx_2_, eae*). The glm function was used in parallel to evaluate the fixed-effects-only model, ignoring individual products. The interaction of whole wheat and organic production was in both cases included, as it was considered biologically plausible. Additionally, although all flour was analysed before the use-by date, a variable splitting the purchase period equally in early/middle/late fixed effect categories was included to ensure storage did not reduce the chance of detection. Fisher’s exact test was used to investigate the association between the presence of *stx* and *eae* in samples. Results were considered significant at *P*<0.05. All data per sample are presented in File S1. The study was not designed to evaluate individual producers, and these were anonymized.

## Results

### Real-time PCR and statistical analysis

Of 200 analysed samples, 24 (12 %) were positive for any *stx* gene by PCR, 13 (6.5 %) each for *stx_1_* and *stx_2_*. Twenty-two (11 %) were positive for intimin (*eae*). The combination of intimin and any *stx* gene occurred in eight samples (4 %), and there was a strong positive correlation between the presence of *eae* and any *stx* gene (Fisher’s exact test *P*=0.0014, OR=5,7). One or more samples from all major brands were found to be positive for *stx* genes. No major differences in the proportion of positive samples were observed overall comparing organic and conventional production, or small and medium/large brands. More *stx*-positive samples were observed from whole grain flour (17 %) compared to white flour (10 %), but none of the investigated explanatory variables were found to be significant by GLMM or GLM analysis. Two samples each were PCR-positive for O157 and O121. No sample was positive for O26. Statistical analysis was not performed on serotype data due to the small number of positive samples.

### Isolation and characterization of EPEC

From the real-time PCR-positive samples, IMS recovered one isolate each of O157 and O121. Both were confirmed by real-time PCR as *stx*-negative but *eae*-positive. Whole-genome sequencing revealed the former of these to be an aEPEC O157:H16 ST10 with several LEE genes, non-LEE encoded effectors, and the putative virulence factors *terC* and *sepA* but negative for *bfp*. The latter isolate was an aEPEC O121:H19/NM ST800. The isolate had a deletion in the gene encoding the H-antigen and is therefore likely phenotypically not H19, although this was not confirmed experimentally. This isolate was also *bfp*-negative and carried virulence genes consistent with a close association with STEC O121:H19/NM but without *stx* genes and *hly*. Full virulence profiles and other data for the recovered isolates are presented in Table 1.

**Table 1.** Isolates of STEC and aEPEC from Swedish wheat flour, with *in silico* determined serotypes, sequence types (seven-gene MLST), shiga toxin subtypes, other virulence factors and ENA accession numbers for sequence data

Isolate	Serotype	Sequence type	Shiga toxin	Other virulence factors	Acc. no.
21-MIK138733	O187:H28	ST200	2g	*astA, celb, ehxA, iss, lpfA, ompT, sta1, terC, traT*	ERS13562958
21-MIK138728	O187:H28	ST200	2g	*astA, cia, celb, ehxA, katP, lpfA, ompT, sta1, terC, traT*	ERS13562957
21-MIK138647	O187:H28	ST200	2g	*astA, celb, ehxA, lpfA, ompT, sta1, terC, traT*	ERS13562953
21-MIK138645	O187:H28	ST200	2g	*astA, celb, ehxA, lpfA, ompT, sta1, terC, traT*	ERS13562952
21-MIK138684	O154:H31	ST1892	1d	*chuA, eilA, mchB,C,F, mcmA, terC, traT*	ERS13562955
21-MIK138717	O154:H31	ST1892	1d	*chuA, eilA, mchB,C,F, mcmA, terC, traT*	ERS13562956
21-MIK138561	O8:H28	ST162	2e	*cba, cma, iss, lpfA, ompT, sta1, terC, traT*	ERS13562949
21-MIK138636	O146:H28	ST738	2b	*astA, hra, iha, ireA, iss, lpfA, mchB,C,F, ompT, subA, terC, usp*	ERS13562951
21-MIK138664	O121:H19/NM*	ST800	None	*astA, cba, cia, cma, eae, efa1, espA,B,F,J, iss, lpfA, nleA,B,C, ompT, tccP, terC, tir, traT*	ERS13562954
21-MIK138611	O157:H16	ST10	None	*eae, espA,B, espF, nleB,C, sepA, terC, tir*	ERS13562950

*H19 with a deletion, see the Results section.

### Isolation and characterization of STEC

A total of 14 samples produced *C*_t_ values <32 for either *stx_1_* or *stx_2_* and were selected for STEC isolation by analysis of colony pools followed by analysis of individual colonies. A total of eight isolates of STEC were recovered, all of which were negative for *eae* and all from different samples. Of the isolates, four were O187:H28 ST200 with *stx_2g_*, the additional enterotoxins *sta1* and *astA*, haemolysin A (*ehxA*) and a handful of other putative virulence factors. Two isolates were O154:H31 ST1892 with *stx_1d_*, and one isolate each was found of O8:H28 ST162 *stx_2e_* and O146:H28 ST738 *stx_2b_*. Full virulence profiles and other data for the recovered isolates are presented in Table 1.

## Discussion

In the present study we show 12 % of Swedish-produced wheat flour samples to be *stx*-positive by real-time PCR after enrichment. Recent studies on cereals in other European countries have reported 11–39% *stx* PCR-positive samples of flour [17–19]. Comparison is complicated by differences in sampling strategies and analysis methods, and in all of these studies some of the positive PCR reactions could be due to the presence of non-viable bacteria or free phages carrying shiga toxin genes [27]. STEC was isolated from eight samples (4 %), but this is likely to underestimate the true prevalence of viable bacteria. The isolation of STEC for which no IMS reagents or selective culture media are available is difficult, and it is highly probable that more isolates would have been found if *C*_t_ thresholds for attempting isolation had been set higher, repeated attempts at analysing colony pools had been performed and if multiple flour aliquots had been analysed, as would have been good practice with a smaller number of samples.

Based on hypothetical routes of contamination, we investigated whether organic or small-scale production could influence the risk of STEC being present in the final product due to differences in farming practices, logistics and processing, but no such effects were observed. It is reasonable to assume that removal of the outer bran of the wheat kernel reduces bacterial contamination [28], and the composition of the microbial flora of whole-grain and refined flour products have indeed been shown to differ [29]. However, although we did find whole-grain products to be *stx*-positive more frequently in the present study, this difference was not significant.

Given the relatively small number of isolates recovered in the present study, it is notable that two STEC serotype/sequence type combinations were found more than once, and most types observed have previously been found in the limited number of studies on flour performed in other countries. This could suggest common routes of contamination or selection for strains more capable of surviving adverse conditions. The most common finding was O187:H28 ST200, which occurred four times in products from four different major brands. The isolates had similar but non-identical virulence profiles, all of which included *stx_2g_* and the heat-stable enterotoxin encoded by the *sta1* gene, which is associated with enterotoxigenic *E. coli* (ETEC). O187:H28 ST200 with the same major virulence factors was also the most common STEC, with a widespread geographical distribution, in German wheat flour in a recent study [30], and has previously been found in retail flour produced in Switzerland [19]. O187:H28 ST200 with *stx_2g_* have also been found in red deer samples collected both in the Italian Alps [31] and in Scotland [32]. We have also recently (2020) isolated a strain with the same combination of traits from a Swedish red deer (authors’ unpublished observations). The second STEC type found more than once was O154:H31 ST1892 carrying the toxin type *stx_1d_*. This type was also repeatedly found in the German flour study previously mentioned [30], and similar STEC strains have been found in Swedish inline milk filters on cattle farms [33]. O146:H28 with *stx_2b_* was also previously observed in the aforementioned German flour study [30], and in Scottish red deer [32]. Isolates matching all three of these combinations of serotypes and shiga toxin types have been reported as causing a small number of sporadic cases of human STEC infection in Sweden without HUS [5, 34].

Isolation of the serotypes that cause the largest number of human STEC and HUS cases is facilitated by the availability of IMS reagents; however, despite this advantage, we found no STEC O157, O121 or O26 in any of the investigated samples. IMS recovered one aEPEC O157:H16 ST10, which was not closely related to STEC O157:H7, and one aEPEC O121:H21/NM ST800, a lineage that has been described as related to STEC O121:H19 but without shiga toxin genes or the O121 virulence plasmid [35]. While these strains are likely capable of causing milder forms of gastrointestinal disease, EPEC is not systematically collected from humans in Sweden and no data are available for comparison. We observed a strong and significant positive correlation between the presence of intimin and shiga toxin genes on the sample level, but no isolates with both traits were recovered. A possible explanation is that faecal contamination is present to a greater extent in some product batches than others, and that this contamination introduces multiple *E. coli* strains. Although intimin-negative STEC are known to cause disease in humans [6], most severe cases reported in Europe are still caused by strains with *eae* together with *stx_2_* [36] and the absence of such strains in the present study thus indicates a lower risk for consumer health.

The sources of STEC in wheat flour found in this and other studies remain to be identified. Irrigation water is unlikely to be a major source, as Swedish wheat fields are rarely watered [37], but manure or contamination from nearby pastures via run-off or dust can potentially transfer STEC from domestic animals. Wildlife feeding on or otherwise occupying the fields also represent a potential source of faecal contamination. Of the areas used for growing cereals in Sweden, 17 % were reported as damaged by wildlife in 2020, with wild boar, cervids and large grazing birds the three most common causes for damage on winter wheat [38]. Post-harvest contamination from birds, rodents or other animals is also plausible. Milling can disperse any faecal material within and between batches of the final product [39], and there is a risk of microbial growth during processing, e.g. in moist residues accumulating inside equipment [28]. The low water activity of the final product will inhibit growth [28], but STEC has been shown to survive in wheat flour for at least 2 years, and it has been suggested that this ability could be a common trait among *E. coli* [40]. We found no effect of storage time on the risk of STEC in a sample, and as our study was conducted on samples from supermarkets the results are likely to reflect consumer exposure.

We note that STEC strains that have been associated with deer in multiple countries have repeatedly been found in European flour. Several studies have reported a high prevalence of STEC in wild ruminants [41], and wheat harvest losses to deer grazing can be significant in areas where the population density is high, as demonstrated with exclusion cages [42]. In general, STEC in wildlife remains an underinvestigated area compared to the extensive work performed on STEC in humans and domestic ruminants, and species associated with crop damage should be prioritized in this regard. Studies to characterize the STEC flora of various wildlife species in Sweden for future source attribution based on typing data are currently ongoing on the national level and in collaboration with European partners and will hopefully shed light on this. Regardless of the yet-to-be-determined sources of the contamination, undercooked products containing wheat flour from any country of origin appear to be a likely source of sporadic cases of STEC infection and a risk for outbreaks in the future.

## Supplementary Data

Supplementary material 1
